# Rapid Fulminant Case of Aspergillus Prosthetic Valve Endocarditis

**DOI:** 10.7759/cureus.1652

**Published:** 2017-09-04

**Authors:** Christian Ortega-Loubon, Manuel Fernández-Molina, Javier Tobar-Ruiz, Nuria Arce-Ramos, Enrique Fulquet-Carreras

**Affiliations:** 1 Cardiac Surgery, Clinic University Hospital of Valladolid; 2 Cardiac Deparment, Clinic University Hospital of Valladolid; 3 Cardiac Department, Clinic University Hospital of Valladolid

**Keywords:** fungal endocarditis, aspergillus flavus, prosthetic valve endocarditis

## Abstract

A 74-year-old male presented to the emergency department 46 days after undergoing an aortic valve replacement. He presented with nonspecific symptoms developed over the previous 15 days, with a new onset of a systolic panfocal murmur. Echocardiography revealed a great vegetation measuring 15 mm by 23 mm causing a severe obstruction of the bioprosthesis. The patient underwent an emergency surgical procedure due to his hemodynamic unsteadiness. During the procedure, we noted an obstruction of the left ventricle outflow tract with pseudoaneurysm of the aortomitral continuity. We debrided the aortic annulus, reconstructed the aortomitral continuity, and replaced the prosthesis, but the patient died. We present a rare fulminant case of Aspergillus endocarditis.

## Introduction

Fungal prosthetic valve endocarditis is a rare but potentially lethal disease. Invasive cardiac Aspergillosis is a severe and extremely life-threatening disease with a few reported cases [1]. We present a case of acute and devastating Aspergillus endocarditis (AE) with an echogenic valvular mass in a male patient who previously underwent an aortic valve replacement. Informed consent was obtained from the patient for this study.

## Case presentation

A 74-year-old male with a history of hypertension and type 2 diabetes underwent an aortic valve replacement for a severe aortic stenosis and bipolar ablation of the pulmonary veins for atrial fibrillation with a normal postoperative course, without the need of a large period of antibiotic therapy, nor the use of indwelling catheters. Forty-six days after hospital discharge, the patient presented to the emergency department hemodynamically stable with asthenia, lethargy, diarrhea, vomiting, and fever for approximately 15 days before presentation, with a new onset of systolic panfocal murmur and swelling of the right ankle.

**Figure 1:**
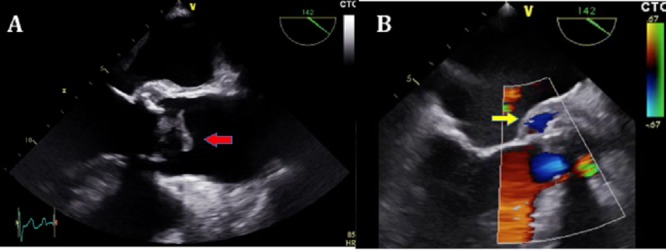
A. Transesophageal echocardiography demonstrating the large vegetation at the aortic bioprosthesis (15 x 23 mm; red arrow), B. Transesophageal echocardiography showing the aortic biological prosthesis and periannular aortic pseudoaneurysm (Doppler flow inside; yellow arrow).

The transesophageal echocardiography revealed a slightly decreased (49%) left ventricle function, aortic biological prostheses with great vegetation of 15 mm by 23 mm; 3.3 cm² anchored to the noncoronary cusp of the prosthesis causing severe obstruction of the prosthesis (peak velocity: 5.8 m/s, mean pressure gradient: 93 mmHg) (Figure 1).

The treatment with gentamicin, vancomycin, and ceftazidime was initiated. However, the patient presented a very torpid evolution, developing cardiogenic shock the following day. The patient underwent an emergency surgical procedure due to his hemodynamic instability and nonsustained ventricular tachycardia (as per the Class I-A recommendations for the management of infective endocarditis). The patient presented cardiorespiratory arrest during the anesthetic induction.

**Figure 2:**
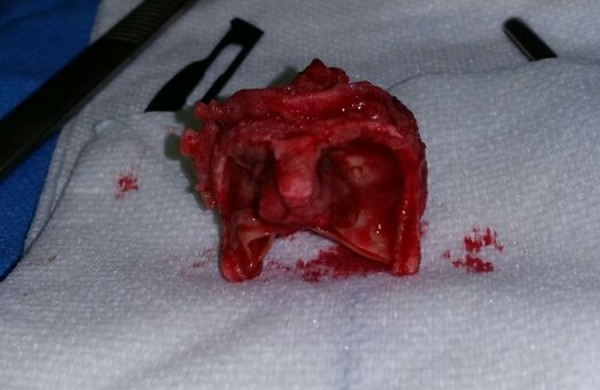
During surgical finding, removed the unstructured and deformed aortic bioprosthesis by large vegetation causing severe aortic stenosis.

We noted an unstructured and deformed aortic bioprosthesis caused by a large vegetation that produced severe aortic stenosis and obstruction of the left ventricle outflow tract, with pseudoaneurysm of the aortomitral continuity (Figure 2).

We cleaned and debrided the aortic annulus and removed the large vegetations, reconstructed the aortomitral curtain with pericardium patch and replaced the aortic bioprosthesis. The patient died during the operation from severe left ventricular dysfunction, extreme vasoplegia, and septic shock. The diagnosis was confirmed during postmortem by a positive culture of the prosthesis for Aspergillus flavus and via multiple negative blood cultures.

## Discussion

Although infrequent, Aspergillus infections can occur in both healthy and immunosuppressed patients. The species Aspergillus fumigatus accounts for 60% to 90% of AE cases, followed by Aspergillus terreus (5% to 20%), with Aspergillus flavus, Aspergillus niger, and Aspergillus nidus accounting for the remaining reported cases [2]. This infection is prevalent in 0.1% of patients with prosthetic heart valves, with the Aspergillus species being responsible for approximately 25% of these cases, second only to a Candida infection [3-5].

Males are more commonly affected than females with a peak incidence during the third and fourth decades of life. Among the risk factors for infective endocarditis in patients undergoing aortic valve replacement, previous valvular surgery is the most determining factor [2-3]. Other factors to consider include prolonged use of antibiotics, intravenous treatment, and use of central catheters [3].

This infection is often the result of contamination during heart surgery [5]. The air is the principal transmission medium for the patients. According to a report by El-Hamamsy, et al., construction or renovation residue near the hospital is a key factor for the occurrence of aspergillosis [5], as in our case, where construction work was taking place near the hospital.

The pathogenesis of AE includes its adherence to the damaged heart valve due to its capacity to bind to the platelet-fibrin matrix. Proteases allow the pathogens to destroy the endothelial lining while protected from the phagocytic activity of mononuclear cells. The avascularity of the heart valve prosthesis and lack of local immune mechanisms create a favorable environment for the pathogens [5].

The AE presents a relative scarcity of peripheral signs of endocarditis. The most common clinical features are fever, major peripheral emboli, and a changing heart murmur. Less frequently, the patients present focal or generalized neurologic deficits, heart failure or dyspnea. This nonspecific clinical presentation explains why AE is rarely suspected and, therefore, diagnosed late with a delayed effective treatment.

As in our case, the AE affects the left-sided valve with the aortic valve more often than the mitral valve.

Aspergillus species are highly angioinvasive with a fast seeding of the vascular wall producing weak areas that lead to an aneurysmal disease, and while urgent surgery is mandatory, it is associated with poor results [5].

The most important part of the treatment of AE is establishing the diagnosis at the right time, a difficult task that hampers therapeutic success. Based on Duke's criteria for the diagnosis of infective endocarditis, a basic criterion is the presence of positive blood cultures. Although this criterion is reliable when referring to bacterial infective endocarditis, the absence of positive blood cultures is not evidenced enough to reject the diagnosis of AE because it is difficult to isolate this microorganism in conventional culture media, and fungemia is intermittent [5]. In our case, blood cultures were negative, but the culture of the bioprosthesis was positive for Aspergillus.

In this case, after a long period when there was no suspicion of endocarditis, the patient’s course was similar to gastroenteritis, but the disease was well established. When the endocarditis was diagnosed, not even an antifungal treatment was set up due to the low diagnostic suspicion. Large vegetations noted on echocardiographic findings deserves greater attention from cardiologists and cardiologists should suspect fungal endocarditis, given the most critical step in this treatment is establishing the diagnosis in a timely fashion.

The AE treatment requires a combination of antifungal therapy and surgical debridement. Radical debridement of necrotic tissue with valve replacement with or without aortic root replacement is the recommended surgical procedure. However, despite the radical surgical strategy, a recurrence rate of up to 40% is observed and has a dismal prognosis. It is not clear when the surgery should be performed [6].

The forecast for AE remains very poor with only around 32% of cases surviving the acute episode. This is due to the immunosuppressed state, diagnostic delay, and embolic complications.

As a teaching point, the surgical indication, in this case, was due to the hemodynamic and electrical instability of the patient. However, the new guidelines recommend urgent surgery for vegetations > 10 mm associated with severe valve stenosis or regurgitation, regardless of the patient's clinical condition.

## Conclusions

Aspergillus endocarditis remains a very rare disease with extremely high lethality with dismal results and unfavorable outcomes mainly because of the delayed diagnosis and the late accurate treatment with little progress in the management of the disease.
